# The Beneficial Endophytic Fungus *Fusarium* *solani* Strain K Alters Tomato Responses Against Spider Mites to the Benefit of the Plant

**DOI:** 10.3389/fpls.2018.01603

**Published:** 2018-11-06

**Authors:** Maria L. Pappas, Maria Liapoura, Dimitra Papantoniou, Marianna Avramidou, Nektarios Kavroulakis, Alexander Weinhold, George D. Broufas, Kalliope K. Papadopoulou

**Affiliations:** ^1^Laboratory of Agricultural Entomology and Zoology, Department of Agricultural Development, Democritus University of Thrace, Orestiada, Greece; ^2^Laboratory of Plant and Environmental Biotechnology, Department of Biochemistry and Biotechnology, University of Thessaly, Larissa, Greece; ^3^Laboratory of Phytopathology, Institute of Olive Tree, Subtropical Plants & Viticulture, Hellenic Agricultural Organization – DEMETER, Chania, Greece; ^4^German Centre for Integrative Biodiversity Research (iDiv) Halle-Jena-Leipzig, Leipzig, Germany; ^5^Institute of Biodiversity, Friedrich Schiller University Jena, Jena, Germany

**Keywords:** endophyte, *Fusarium*, gene expression, performance, spider mites, tomato, volatiles

## Abstract

Beneficial microorganisms are known to promote plant growth and confer resistance to biotic and abiotic stressors. Soil-borne beneficial microbes in particular have shown potential in protecting plants against pathogens and herbivores via the elicitation of plant responses. In this study, we evaluated the role of *Fusarium solani* strain K (FsK) in altering plant responses to the two spotted spider mite *Tetranychus urticae* in tomato. We found evidence that FsK, a beneficial endophytic fungal strain isolated from the roots of tomato plants grown on suppressive compost, affects both direct and indirect tomato defenses against spider mites. Defense-related genes were differentially expressed on FsK-colonized plants after spider mite infestation compared to clean or spider mite-infested un-colonized plants. In accordance, spider mite performance was negatively affected on FsK-colonized plants and feeding damage was lower on these compared to control plants. Notably, FsK-colonization led to increased plant biomass to both spider mite-infested and un-infested plants. FsK was shown to enhance indirect tomato defense as FsK-colonized plants attracted more predators than un-colonized plants. In accordance, headspace volatile analysis revealed significant differences between the volatiles emitted by FsK-colonized plants in response to attack by spider mites. Our results highlight the role of endophytic fungi in shaping plant–mite interactions and may offer the opportunity for the development of a novel tool for spider mite control.

## Introduction

Plants have evolved sophisticated mechanisms to defend themselves against biotic stressors such as pathogenic microorganisms and herbivorous arthropods. In particular, the ways plants respond to herbivory involve the expression of direct defenses such as toxins and anti-digestive proteins that target the herbivore but also indirect defenses to attract the natural enemies of the attacker to the plant via, for example, the emission of herbivore-induced plant volatiles itself (Karban and Baldwin, 1997; Schaller, 2008; Dicke and Baldwin, 2010). Direct and indirect defenses can be constitutively produced and/or specifically induced after attack (Karban and Baldwin, 1997; Erb et al., 2012). For example, many defense mechanisms are initiated upon recognition of the attacker after which downstream defense signaling is activated leading to, for example, the production of defensive compounds that negatively affect the attacker (Wu and Baldwin, 2010). The phytohormones jasmonic acid (JA) and salicylic acid (SA), ethylene (ET) and abscisic acid (ABA) are key regulators in plant defense against herbivores, modulating afterwards the expression of defense-related genes and the production of defensive compounds (Erb et al., 2012; Pieterse et al., 2014). Importantly, cross-talk among the phytohormonal pathways (e.g., antagonistic relationships between the JA and SA pathways) allows plants to fine-tune their defensive responses depending on the organisms encountered in a multi-species environment (Pieterse et al., 2012).

Plant defense production is generally assumed to be a costly process that requires the allocation of valuable resources to resistance at the expense of growth and reproduction (Cipollini et al., 2003; Walters and Heil, 2007; Pappas et al., 2017). In addition to physiological costs, i.e., those related to energy investment, ecological costs, such as the disturbance of plant interactions with other organisms (Agrawal et al., 1999; Thaler et al., 1999; Ballhorn et al., 2014; Ohm and Miller, 2014), both, may ultimately result in reduced plant performance (Herms and Mattson, 1992). To minimize plant defense related costs, the majority of defenses are activated after herbivore attack only. Besides energy savings, defense induction may also protect plants from auto-toxicity and, importantly, allows tailoring of plant responses against specific attackers (Baldwin and Callahan, 1993; Pappas et al., 2017). In addition, defense priming, a physiological state of readiness that takes place after initial exposure to a stressor that prepares plants for a subsequent stress, is an additional strategy that plants have evolved against herbivory (Heil and Kost, 2006; Frost et al., 2008). Eventually, primed plants are able to respond faster, stronger and thus more effectively to certain attackers compared to non-primed plants, often at a lower cost to the plant (Martinez-Medina et al., 2016).

Priming of defenses can occur after initial exposure of plants to harmful herbivores or pathogens but also, when plants are exposed to beneficial non-pathogenic organisms. Selected root-colonizing microbes (e.g., bacteria and fungi) have long been recognized for their ability to antagonize soil-borne pathogens, facilitate nutrient uptake, improve plant growth, and also prime the plant immune system against aboveground future attackers in return for carbohydrates secreted by the plant (Smith and Smith, 2011; Pineda et al., 2013; Pieterse et al., 2014; Finkel et al., 2017). For example, defense priming by plant-growth promoting rhizobacteria (PGPR), generally referred to as induced systemic resistance (ISR), is characterized by increased acceleration of defense-related genes upon herbivore and pathogen attack and generally known to be JA-regulated, not shown to trade-off with plant fitness (Rosenblueth and Martínez-Romero, 2006; Van Wees et al., 2008). In addition, other microbes such as plant-growth promoting fungi (PGPF) and arbuscular mycorrhizal fungi (AMF) have been shown to variously impact herbivorous arthropods on aboveground plant parts. As such, soil-borne beneficial microbes are of particular interest as ‘vaccination’ agents, capable of enhancing plant resistance to biotic stressors most possibly without compromising crop production.

Mechanisms involved in plant defense induction by beneficial soil microbes mediate both direct and indirect responses against aboveground herbivores (Pineda et al., 2010; Rasmann et al., 2017; Shikano et al., 2017). Microbe-ISR can be directly effective against insects and mites because it involves an increased sensitivity to JA (Rosenblueth and Martínez-Romero, 2006; Van Wees et al., 2008). Thus, chewing herbivores but also phloem feeders (e.g., aphids, whiteflies), that normally counteract JA-defenses via crosstalk, can be negatively impacted by JA-mediated plant responses induced by beneficial microbes (Pineda et al., 2010). In addition, such plant-mediated effects have been shown to not only depend on the microbe group (e.g., PGPR or AMF) but also on the feeding specialization of the herbivore. For example, AMF are believed to show negative effects against generalists and mesophyll feeders and positive or neutral effects on specialist chewers and phloem feeders (Hartley and Gange, 2009; Pineda et al., 2010; Shikano et al., 2017). On the other hand, JA is also involved in indirect defense responses against herbivores and thus it is reasonable to expect that microbe-ISR is capable of altering the composition or the emission rate of the volatile blend emitted by microbe-colonized plants in response to herbivory (Pineda et al., 2010; Rasmann et al., 2017). Indeed, selected soil-borne microbes have been shown to modify the volatile blends thereby increasing the attractiveness of the infested plants to the natural enemies of the attacker (e.g., Fontana et al., 2009; Schausberger et al., 2012; Pineda et al., 2013). Whether both direct and indirect defenses of a plant against a particular herbivorous species can be affected by a single microbe species via ISR remains largely unknown.

Despite the vast diversity of soil-borne beneficial microbes that are associated with plants, much of our current knowledge about microbe-ISR effects on herbivores derives from studies on two microbial groups mainly, PGPR and AMF. Nevertheless, a number of diverse endophytic fungi are known to also inhabit roots, forming variable associations with the plants, ranging from parasitic to mutualistic, without, however, causing apparent disease symptoms in plants (Wilson, 1995; Schulz and Boyle, 2005; Hartley and Gange, 2009; Rodriguez et al., 2009). In contrast to AMF, the ecological roles of the most common endophytic fungi, especially those that are horizontally transmitted via spores (e.g., Ascomycetes), currently remain elusive although generally believed to also play an important role in plant protection against herbivores (Jaber and Vidal, 2009; Rodriguez et al., 2009; Gan et al., 2017). Indeed, certain root endophytic fungi have been shown to increase the expression of defense-related genes and the production of secondary metabolites that may be relevant to plant defense (Pieterse et al., 2014). In addition, a few studies involving endophytic fungi have reported negative effects on above ground herbivores thus enhancing their potent role in plant resistance to biotic stressors (Jallow et al., 2004; Jaber and Vidal, 2009, 2010; Muvea et al., 2014; Coppola et al., 2017; Contreras-Cornejo et al., 2018). Nevertheless, our understanding of endophytic fungi – plant – herbivore interactions is still at its infancy thus calling for more empirical studies on the significance of horizontally transmitted endophytes in plant–herbivore interactions (Gan et al., 2017).

In this study, we hypothesized that tomato responses to spider mites can be enhanced by soil-borne beneficial microbes, particularly endophytic fungi. Spider mites are mesophyll cell-content feeders and many species are major pests in agriculture. Specifically, the two-spotted spider mite, *Tetranychus urticae* Koch (Acari: Tetranychidae) is a cosmopolitan species that infests a high number of crops belonging to different plant families. In tomato, *T. urticae* induces JA and SA defenses simultaneously and has been shown to be highly sensitive to JA-mediated defenses (Kant et al., 2008; Alba et al., 2015; Ataide et al., 2016). Besides direct defense responses, tomato also activates volatile production in response to *T. urticae* feeding. This results in spider mite-infested plants being highly attractive to its natural enemies, such as the predatory mite *Phytoseiulus* *persimilis* Athias-Henriot (Acari: Phytoseiidae) (Kant et al., 2004). To the best of our knowledge, plant-mediated effects of soil-borne microbes on spider mites have been scarcely addressed so far, mainly for AMF (Hoffmann et al., 2009, 2011; Schausberger et al., 2012; Khaitov et al., 2015), and no study has ever dealt with beneficial root endophytic fungi in tomato.

We thus, assessed the impact of the endophytic fungus *Fusarium solani* strain K (FsK) on the performance of *T. urticae* on tomato and recorded the changes in defense-related gene expression on FsK-colonized compared to control plants. Furthermore, we analyzed the volatile blends emitted from FsK-colonized and control plants and recorded the responses of the zoophytophagous predator *Macrolophus pygmaeus*, a natural enemy of spider mites, toward these plants. *Fusarium solani* strain K is an horizontally transmitted endophytic fungal isolate that colonizes tomato roots, including vascular tissues to the crown area, without fungal growth progressing to aboveground tissues (Kavroulakis et al., 2007). In tomato, FsK is known to confer ethylene-dependent resistance against fungal root and foliar pathogens (Kavroulakis et al., 2007). In addition, FsK-colonized plants are more resistant to plant damage caused by the zoophytophagous predator *Nesidiocoris tenuis*, possibly via the JA and/or ethylene signaling pathways (Garantonakis et al., 2018). We thus, hypothesized that FsK may be effective against herbivores too and included spider mites in our experiments to first, assess FsK potential on impacting spider mite performance but also, to explore putative mechanisms involved in FsK-tomato-spider mite interactions.

## Materials and Methods

### Plants and Growing Conditions

Tomato [*Solanum* *lycopersicum* L., cv. Ace 55 (Vf)] plants were used in all experiments as well as in herbivore and predator rearing. Experimental plants were grown from seeds that were surface-sterilized in 2.5% NaOCl and sown directly in pots (Ø 12 cm), each containing approximately 300 cm^3^ of a sand mixture with vermiculite (2:1) and a N-P-K fertilizer (20–20–20) to a total concentration of 0.8 gl^-^1 of potting mix. Plants used for rearing arthropods were grown from seeds in pots (Ø 12 cm) with soil (Klasmann-TS2). All plants were maintained in separate climate chambers (25 ± 2°C, 16:8 LD, 60–70% RH) and watered every other day and once a week fertilized. Experimental plants were fertilized with a balanced nutrient solution (Hoagland 100%) and a N-P-K fertilizer (20–20–20) was used to fertilize plants grown to rear arthropods. Plants used in the experiments were 4–5 weeks old.

### Fungal Strain, Plant Inoculation and Quantification of Fungal Colonization

Experimental tomato plants were inoculated with the endophytic non-pathogenic *F. solani* strain FsK (Kavroulakis et al., 2007) routinely cultured on potato dextrose broth (PDB) at 25°C for 5 days in the dark. Following removal of mycelium fragments by sieving, conidia were recovered by centrifugation at 4000 *g*, counted using a haemocytometer and suspended in an appropriate volume of 0.85% NaCl in order to achieve the desired inoculum concentration. Application of the inoculum of strain FsK with 10^2^ conidia cm^-3^ of potting mix was performed as water drench 1 week after seed sowing. FsK colonization was verified with destructive sampling of 10 plants per batch and treatment (FsK-colonized and control plants) for all experiments by PCR 2 weeks after seed sowing and colonization levels were estimated 4 days after spider mite infestation by means of qPCR.

Samples were used for whole genomic DNA extraction using the “NucleoSpin^®^ Plant II genomic DNA extraction” kit (MACHEREY-NAGEL GmbH & Co. KG, Duren, Germany). FsK colonization of root tissues after infestation and spider mite feeding was assessed via qPCR by using primers pair for a ca 170 bp fragment of the *Nectria haematococca* translation elongation factor 1a (*Tef*-*1a*) gene (Supplementary Table S1). An external standard curve was generated in order to quantify the copy number of *Tef*-*1a* gene in total DNA extracted from root tissues of FsK-colonized plants. The standard curve was generated as follows: *Tef*-*1a* gene was amplified using FsK genomic DNA as template, the PCR product was purified and ligated into pGEM-T Easy vector (Promega, Madison, United States) and transformed to competent *Escherichia coli* DH5a cells. The recombinant plasmid was extracted again (NucleoSpin Plasmid, Macherey Nagel) and its concentration was determined via Qubit 3.0 Fluorometer. The copy numbers of the targeted gene were calculated from the concentration of the extracted plasmid DNA. Amplification occurred in a 10 μl reaction mixture containing Kapa SYBR FAST qPCR Master Mix (1x) Universal, 200 nM of each primer, and 1 μl of DNA, using the following thermocycling protocol: 3 min at 95°C; 45 cycles of 15 s at 95°C, 20 s at 58°C followed by a melting curve to check the specificity of the products. PCR products were further analyzed on a 1.5% agarose gel in order to check for potential non-targeted amplifications. Data were analyzed using the Student’s two-tailed homoscedastic *t*-test to compare the colonization of FsK with the +/- spider mite-infested group.

### Herbivore and Predator Rearing

Spider mites (*T. urticae*) were reared on detached tomato leaves on wet cotton wool inside plastic trays that were kept in a climate room at 25 ± 2°C, 16:8 LD, 60–70% RH. Fresh tomato leaves were provided every 3 days and the trays were filled with water to maintain leaf vigor. In this study, we used the ‘KOP’ spider mite line kindly provided by Dr. Merijn Kant (University of Amsterdam). This is a tomato-adapted line previously shown to resist JA defenses in tomato (Ament et al., 2004; Kant et al., 2008). For all experiments, adult female mites (2–4 days old) were used. These were obtained by infesting tomato plants with a high number (approximately 200) of female spider mites that were allowed to lay eggs for 48 h at 25 ± 2°C, 16:8 LD. The next day, the adult mites were carefully removed and the plants were maintained at the same conditions till adult spider mites emerged (after approximately 16 days).

*Macrolophus* *pygmaeus*, a zoophytophagous predator that feeds both on prey and plant was reared on young tomato plants (2-weeks old) in plastic cages (47.5 cm × 47.5 cm × 47.5 cm, BugDorm MegaView Science Co., Ltd.) maintained at 25 ± 2°C, 16:8 LD, 60–70% RH, as described by Pappas et al. (2015). The rearing was established with adults of the commercially available product MIRICAL (Koppert B.V. Berkel en Rodenrijs, Netherlands). Bee pollen and eggs of *Ephestia* *kuehniella* were provided *ad libitum* as supplementary food for the predators. For the olfactometer experiments, we used young female predators (7–10 days old) that were obtained by allowing 5 predator females to lay eggs on young tomato plants for 48 h. Emerging nymphs were fed with *E. kuehniella* eggs sprinkled on tomato plants until adulthood.

### Herbivore Performance and Feeding Damage

Spider mite performance on tomato plants that were colonized by the endophyte was assessed by infesting FsK-colonized and control (un-colonized) tomato plants with 45 female spider mites per plant on 3 leaflets [15 females per leaflet, leaflets were selected as described by Alba et al. (2015)] for a period of 4 days. Subsequently, the number of eggs and live females per plant were recorded. Spider mites were prevented from escaping by a lanolin circle applied around the petiolule of each leaflet. Thirteen plants from two independent experiments were used per treatment.

Feeding damage inflicted by spider mites on FsK- colonized and control plants was recorded after 10 days when 10 female spider mites per leaflet (3 infested leaflets, thus 30 females per plant) had been feeding on tomato plants, as described above. Eight plants from two independent experiments were used per treatment. All spider mite-infested leaflets were collected and scanned digitally. Leaf area damage was assessed as described by Cazaux et al. (2014).

Means (number of surviving spider mites, number of eggs, feeding damage) were compared by Student’s *t*-test (SPSS, 2011). Shapiro–Wilk test was used to verify the normality of error distribution.

### Plant Growth Parameters

To assess the extent to which plant growth parameters (root and shoot biomass) are affected by fungus colonization and/or spider mite infestation, 4–5 weeks old FsK-colonized and control (un-colonized) tomato plants were infested with 30 female spider mites (10 females per leaflet, 3 leaflets per plant) for a period of 10 days. Subsequently, plant shoot tissue was harvested and weighed on a microbalance. In addition, roots were harvested, cleaned in water, dried on tissue paper and weighed. Four treatments in total were included in this experiment: FsK-colonized plants (+F/-T), FsK-colonized and spider mite infested plants (+F/+T), un-colonized and spider mite-infested plants (-F/+T) and clean plants (-F/-T). Eight plants from two independent experiments were used per treatment. Differences in shoot and root weight among treatments were analyzed by two-way analysis of variance (ANOVA) followed by Tukey’s HSD *post hoc* tests (*P* < 0.05). Prior to data analysis Shapiro–Wilk and Levene’s tests were used to verify the assumptions of parametric analysis, i.e., normality of error distribution and equality of variances, respectively (R Core Team, 2016).

### Tomato Defense-Gene Expression

FsK-colonized and control tomato plants (4–5 weeks old) were infested with 45 spider mites, as described above for the performance experiments. Another set of FsK-colonized and control plants received no spider mite treatment. This experiment was conducted in a climate room at 25 ± 2°C, 16:8 LD and 60–70% RH. After 4 days of spider mite feeding, infested leaflets as well as leaflets of the same position on uninfested plants, were harvested, flash frozen on dry ice and stored at -60°C until mRNA extraction (*n* = 6 biological replicates per treatment). The three leaflets harvested from the same plant were pooled to form one biological replicate. The experiment was repeated with the same experimental set-up one month later.

To explore tomato defenses, we analyzed the expression of the following genes: *JIPI*-*21*, *WIPI*-*II*, *PI*-*IIc*, *PPO*-*D*, *PPO*-*F*, *LOXD*, *PR*-*1A*, *PR*-*P6*, *GGPS1*, *GLU*-*A*, *GLU*-B, *CHI3*, and *CHI9*. RNA was extracted from plant tissues using a LiCl protocol according to Brusslan and Tobin (1992). RNA samples were treated with DNase I from Thermo Scientific as follows: samples were incubated in 37°C for 30 min, the tubes were transferred on ice and 1 μl EDTA 50 mM was added before the inactivation of the DNase at 65°C for 10 min. In order to ensure no genomic DNA was left, a PCR was performed using primers specifically designed to amplify the tomato housekeeping gene *ubiquitin*. cDNA was made with a 1st-strand cDNA synthesis kit from TAKARA using an oligo-dT primer according to the manufacturer’s instructions. Quantitative PCR was conducted with a SYBR-Fast kit from Kappa Biosystems according to the manufacturer’s instructions on a Bio-Rad CFX Connect Real Time thermo-cycler. The sequences of gene-specific primers used in RT-PCR analysis are shown in Supplementary Table S1. The resulting first-strand cDNA was normalized based on expression of the housekeeping gene *ubiquitin* (*UBQ*). Analysis was carried out as described in Delis et al. (2011) using the geometric mean of *ubiquitin* as reference gene. To calculate the fold-change in transcript levels, the relative expression of each target gene was calculated for each sample as described, and the ratio of each transcript’s relative expression was normalized to its expression in control samples. Differences in gene expression between treatments were analyzed by two-way analysis of variance (ANOVA) followed by Tukey’s HSD *post hoc* tests (*P* < 0.05). Data were tested for normality using the Shapiro–Wilk test (SPSS, 2011). Results of the two independent experiments are presented in Figures 4, 5, Supplementary Table S2 and Supplementary Figure S2. For the visualizations, data manipulation was performed in RStudio with the pheatmap package (version 1.0.8) (R Core Team, 2016; Kolde, 2018). Sample/gene grouping was based on hierarchical clustering (complete linkage algorithm) of the Euclidean sample/gene distances of the differentially expressed genes detected by ANOVA (Tukey’s *post hoc* tests, *P* < 0.05).

### Headspace Collection and Analysis of Tomato Volatiles

The collection of volatile organic compounds (VOCs) was performed at 25 ± 1°C and 60–70% RH between 10:00 am and 17:00 pm for treatments (1)–(5) mentioned below in ‘Olfactometer Assays’ (5–6 biological replicates in total per treatment) with a push-pull dynamic volatile collection system. The system consisted of 5 L glass chambers each containing one tomato plant. Pots were wrapped in aluminum foil to avoid trapping soil and plastic volatiles. Five independent chambers containing randomly assigned plants were run simultaneously. Charcoal-filtered, humidified air was pumped in the containers at a rate of 1 L/min and pull out at 0.6 L/min passing through stainless steel tubes loaded with 200 mg of Tenax (MARKES, Llantrisant, United Kingdom). The sampling duration was adjusted to 30 min. In addition, we also sampled volatiles from empty glass chambers. Those “air blanks” were used in the further data processing to exclude systemic contamination compounds.

Tomato volatiles were analyzed by a thermal desorption-gas chromatograph-mass spectrometer (TD-GC-MS) consisting of a thermodesorption unit (MARKES, Unity 2, Llantrisant, United Kingdom) equipped with an autosampler (MARKES, Ultra 50/50). Tubes were desorbed with helium as carrier gas and a flow path temperature of 150°C using the following conditions: Dry Purge 5 min at 20 ml/min, Pre Purge 2 min at 20 ml/min, Desorption 8 min at 280°C with 20 ml/min, Pre Trap fire purge 1 min at 30 ml/min, Trap heated to 300°C and hold for 4 min. The VOCs were separated on a gas chromatograph (Bruker, GC-456, Bremen, Germany) connected to a triple-quad mass spectrometer (Bruker, SCION). Separation took place on a DB-5MS column (30 m × 0.25 mm × 0.25 μm. Restek, Germany). The conditions of the GC were as follows: 40°C for 5 min, 5°C/min to 185°C, 30°C/min to 260, and hold for 0.5 min. The mass spectrometer was operated in full scan mode with the following parameters: transfer line temperature 280°C, ion source temperature 260°C, scan time 250 ms, scan range 40–550 m/z, ionization 70 eV.

We selected the most prominent peaks in the chromatograms (signal to noise ratio > 10). Peaks that were also present in air blanks were regarded as systemic contamination and were excluded from further analysis. This procedure resulted in 41 compounds. The peak areas of these compounds were calculated using the Bruker Workstation software (v8.0.1). PCA analysis was performed on autoscaled data in R (v3.3.2) (R Core Team, 2016) using the packages ggplot2 (v2.2.1) (Wickham, 2009) and ggfortify (v0.4.4) (Tang et al., 2016). Differences in the emissions of the selected compounds were analyzed by two-way analysis of variance (ANOVA) followed by Tukey’s HSD *post hoc* tests (*P* < 0.05). Prior to data analysis Levene’s tests was used to verify the assumption of equality of variances (R Core Team, 2016).

### Olfactometer Assays

To assess the extent that the volatile blend emitted by clean or spider mite-infested plants may be changed by FsK-colonization and eventually, the responsiveness of the mirid predator *M. pygmaeus* to these plants, we performed a series of vertical Y-tube olfactometer assays, as described by Lins et al. (2014). The Y-tube olfactometer (4.0 cm diameter, main arm 20 cm long, side arms 23 cm long, 75° angle between the side arms) was connected to a volatile collection system. Each side arm of the olfactometer was connected to a 4 L glass vessel containing one tomato plant. Each pot was wrapped with aluminum foil to restrict the emission of soil/plastic volatiles. Pressurized air was purified by passing through a wash bottle filled with activated charcoal pellets, humidified and entered the odor chambers at a rate regulated by means of a flowmeter of 2 L/min. From the outlet port at the top of the odor chamber the air was led to the arms of the olfactometer. At the base of the Y-tube the air was sucked off by means of a vacuum peristaltic pump, producing an air flow of 0.4 L/min in each side arm and 0.8 L/min in the base of main arm of the Y-tube. Teflon tubing was used for the connections between different parts of the set-up.

One predator female (5–7 days old) was introduced into the main arm of the olfactometer and allowed to make a choice between the two arms, i.e., volatile sources. Each female was considered to have made a choice when covering more than 12 cm inside each chosen arm. The females that did not make a choice within 10 min were excluded from data analysis. Each predator was used only once and had no visual contact with the plants during the bioassay since they were separated with a white panel. Before the bioassays the predators were starved for 24 h. We recorded 67–70 replicates (individuals) depending on the treatment (i.e., odor) combination. Every two replicates, the olfactometer side arms were switched to exclude positional effects. Every 10 female predators the Y-tube and the glass vessels were washed with ethanol (70%) and neutral soap and were allowed to dry before use. Olfactometer assays were performed in a room at 25 ± 1°C and 60–70% RH between 10:00 am and 17:00 pm. Predator responses were assessed for combinations of the following treatments: (1) FsK-colonized plants (+F/-T), (2) spider mite-infested, un-colonized plants (-F/+T), (3) FsK- colonized, spider mite-infested plants (+F/+T), (4) clean plants (-F/-T), (5) clean air (blank, i.e., no plant). For the Y-tube olfactometer bioassays, the null hypothesis that females of *M.* *pygmaeus* showed no preference for either arm of the olfactometer (i.e., 50:50 response) was tested using χ^2^ test (SPSS, 2011).

## Results

### Spider Mite Performance, Fungal Colonization and Feeding Damage

**FIGURE 1:**
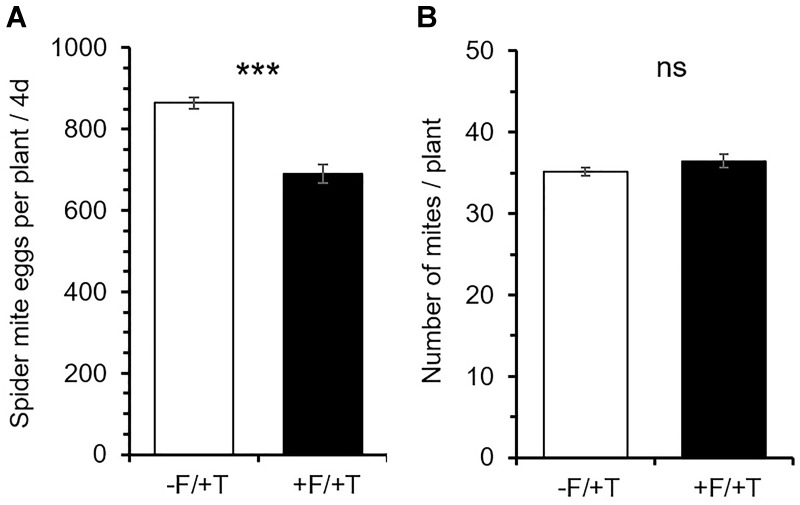
Effects of tomato colonization by the endophyte *Fusarium solani* strain K (FsK) on spider mite (*Tetranychus urticae*) performance. One week after seed sowing plants were either inoculated with the endophyte (black bars, +F) or not (white bars, –F). Subsequently, plants were infested with 45 spider mites (15 mites per leaflet, 3 leaflets per plant, +T) when 4–5 weeks-old for a period of 4 days. Mean ± SE (*n* = 13) of the **(A)** number of spider mite eggs per plant and **(B)** number of live adult females per plant recorded on FsK-colonized and control plants after 4 days. Significant differences between treatments are indicated by asterisks after Student’s *t*-test: ^∗∗∗^*P* < 0.001, ns, not significant.

FsK-colonization affected spider mite performance with the number of eggs recorded on leaves of colonized plants within 4 days being significantly less than those on control (un-colonized) plants [Figure 1A; *t*_(24)_ = -6.527; *P* < 0.001]. In contrast, FsK-colonization did not affect the number of mites found alive on these compared to untreated control plants [Figure 1B; *t*_(24)_ = 1.376; *P* = 0.182]. Notably, spider mite infestation had no effect on FsK colonization compared to non-infested un-colonized plants [Supplementary Figure S1; *t*_(10)_ = 0.179; *P* = 0.861].

**FIGURE 2:**
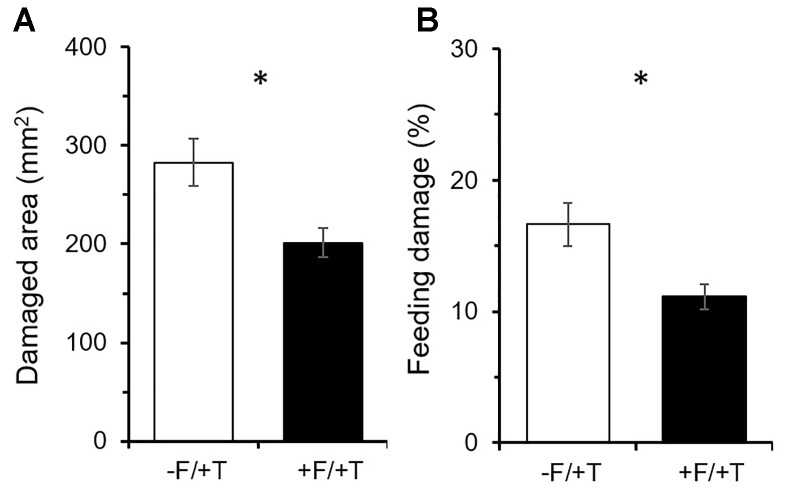
Effects of tomato colonization by the endophyte *Fusarium solani* strain K (FsK) on the feeding damage inflicted by spider mites (*Tetranychus urticae*). One week after seed sowing plants were either inoculated with the endophyte (black bars, +F) or not (white bars, –F). Subsequently, plants were infested with 30 spider mites (10 mites per leaflet, 3 leaflets per plant, +T) when 4–5 weeks-old for a period of 10 days. Mean ± SE (*n* = 8) of the **(A)** damaged area per plant and **(B)** proportion of damaged to total leaflet area inflicted by spider mites on FsK-colonized compared to control plants over a period of 10 days. Significant differences between treatments are indicated by asterisks after Student’s *t*-test: ^∗^*P* < 0.05.

Tomato colonization by FsK had a significant effect on the damage inflicted by spider mites over the 10 days of feeding, which was reduced on colonized compared to un-colonized plants [Figure 2A; *t*_(14)_ = 2.91; *P* < 0.05]. Total leaflet area was similar between FsK-colonized and control plants [*t*_(14)_ = 0.63; *P* = 0.535] and feeding damage was reduced by approximately 28.7% resulting in a significant decrease in the proportion of damaged to total leaflet area compared to control plants [Figure 2B; *t*_(14)_ = 2.89; *P* < 0.05].

### Shoot and Root Biomass

**FIGURE 3:**
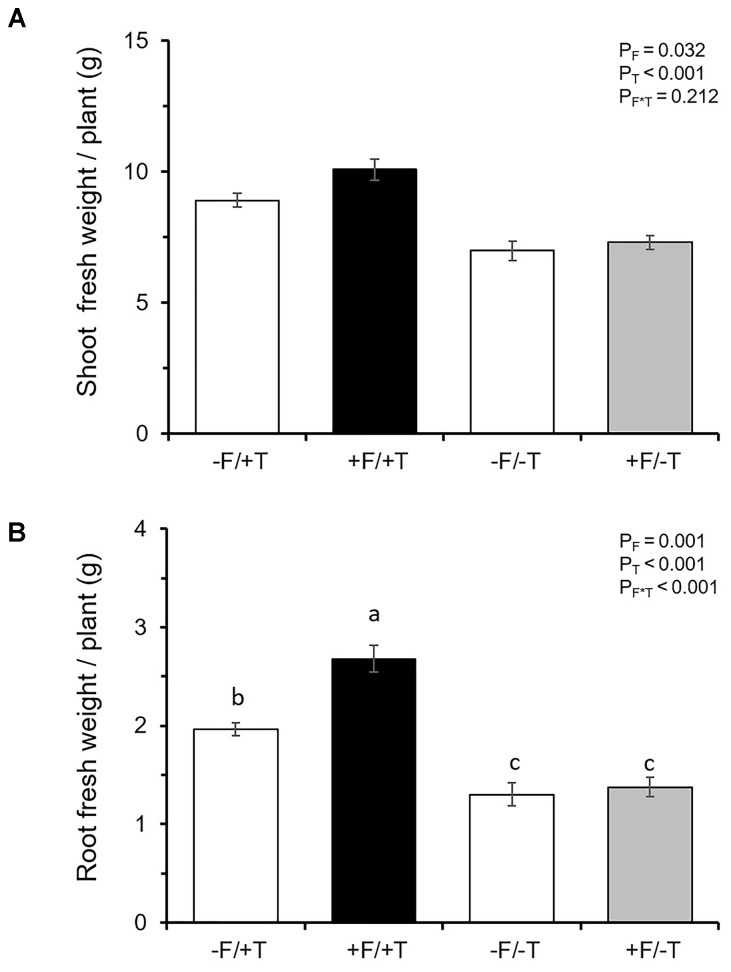
Effects of spider mite infestation and tomato colonization by the endophyte *Fusarium solani* strain K (FsK) on plant growth parameters. One week after seed sowing plants were either inoculated with the endophyte (black and gray bars, +F) or not (white bars, –F). Subsequently, plants were either infested with 30 spider mites (10 mites per leaflet, 3 leaflets per plant, +T) or not (–T) when 4–5 weeks-old for a period of 10 days. Mean ± SE (*n* = 8) of **(A)** shoot and **(B)** root fresh weight per plant across all treatments. Significant differences between treatments are indicated by different letters by Tukey’s *post hoc* tests after ANOVA: *P* < 0.001.

We tested whether tomato colonization by the endophyte would affect plant growth parameters of spider mite-infested or control (non-infested) plants. We found a significant endophyte effect (*F*_1,28_ = 5.084, *P* = 0.032), a highly significant herbivore effect (*F*_1,28_ = 50.178, *P* < 0.001) and no significant interaction effect (*F*_1,28_ = 1.632, *P* = 0.212) on shoot fresh weight. Spider mite-infested plants were heavier compared to control (non-infested) plants and FsK-colonization significantly affected shoot fresh weight (Figure 3A). In addition, two-way ANOVA revealed a significant endophyte effect (*F*_1,28_ = 13.372, *P* = 0.001), a highly significant herbivore effect (*F*_1,28_ = 82.266, *P* < 0.001) and a significant interaction effect (*F*_1,28_ = 8.796, *P* = 0.0061) on root fresh weight. Roots of spider mite-infested plants were heavier and this effect was significantly enhanced when these plants were also colonized by FsK (Figure 3B).

### Tomato Defense-Gene Expression

To assess the effects of FsK-colonization on defense-gene expression in response to spider mite feeding, we used well-established defense marker genes that are known to mark mite-activated JA and SA defenses in tomato (Li et al., 2002; Ament et al., 2004; Kant et al., 2004, 2008; Alba et al., 2015; Martel et al., 2015). In addition, our study included the genes *GLU-A* and *GLU-B*, *CHI3* and *CHI9*, typically induced against fungi or other herbivores [e.g., whiteflies, Puthoff et al. (2010)].

**FIGURE 4:**
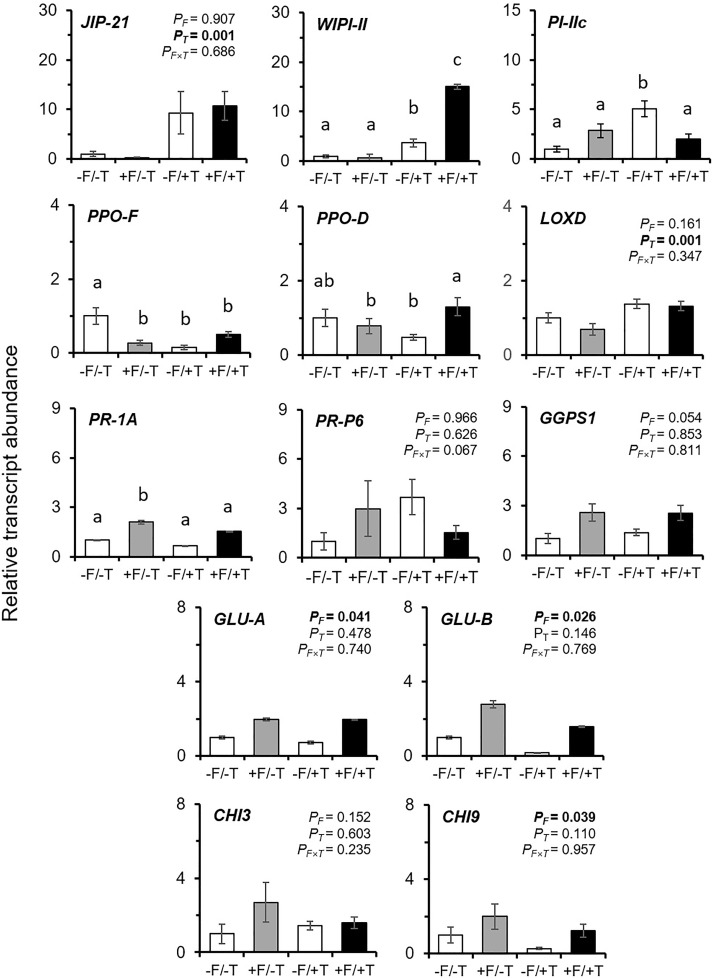
Effects on the transcript levels of defense marker genes in tomato plants colonized by the endophyte *F. solani* strain K (+F) and/or infested with spider mites (*T. urticae*, +T) compared with the untreated control (–F/–T). Values are the average ± SE of two technical replicates for each of six biological replicates of an independent experiment (Supplementary Table S2). Expression levels for all target genes were normalized to the geometric mean of ubiquitin and actin expression levels in each sample as a reference. Two-way ANOVA (α = 0.05) *P*-values are shown for each gene on each panel in the case of non-significant interaction effects (*P_F^∗^T_* > 0.05) and significant *P*-values (<0.05) are indicated in bold: *P_F_*_,_ probability value for the endophyte (FsK) effect; *P_T_*, probability value for the herbivore (T) effect; *P_F^∗^T_*, probability value for FsK × T interaction. In the case of significant interaction (*P_F^∗^T_* < 0.05) significant differences between treatments are indicated by different letters by Tukey’s *post hoc* tests after two-way ANOVA: *P* < 0.05. *JIP-21*, *WIPI-II*, *PI-IIc*, *PPO-F/D*, and *LOXD* are JA-marker genes; *PR-1A* and *PR-P6* are SA-marker genes; *GLU-A/B* and *CHI3/9* are fungal (or herbivore, e.g., whitefly)-marker genes; *GGPS1* has been reported as responsive to spider mite infestation in tomato (see relevant references in manuscript).

**FIGURE 5:**
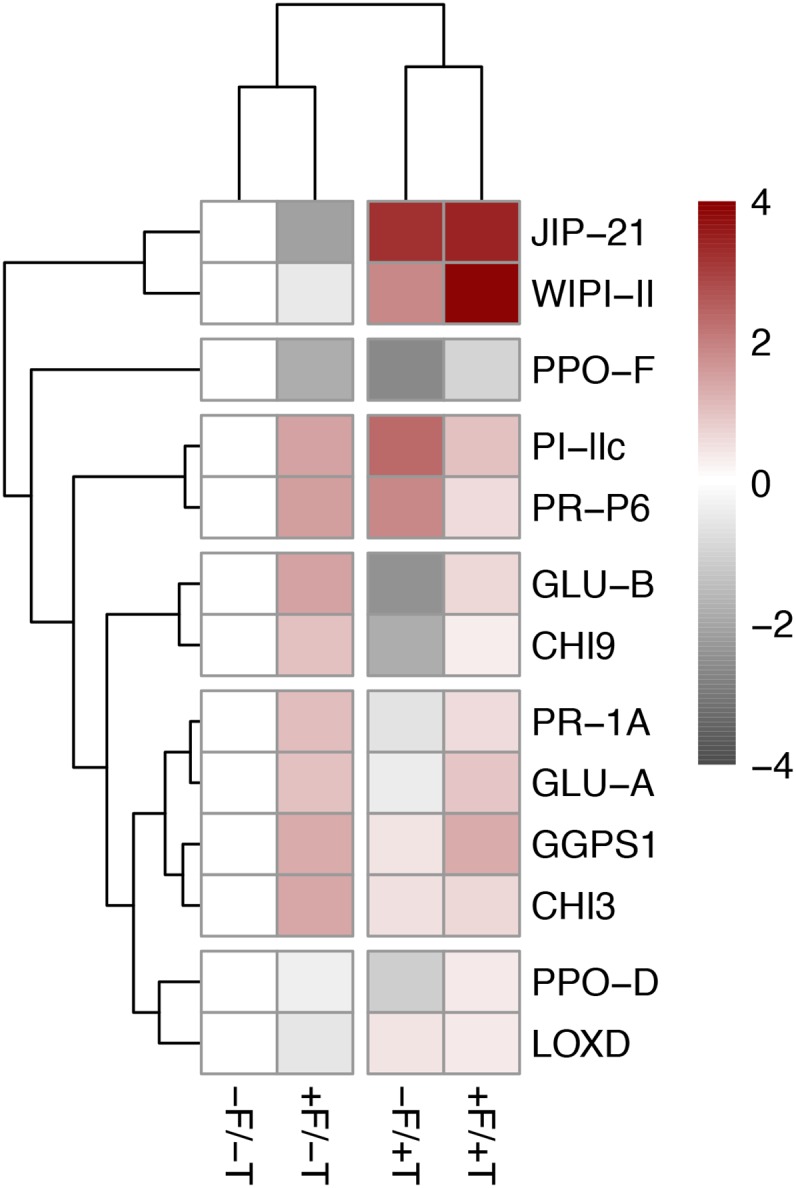
Heatmap of the differentially expressed plant defense genes in tomato plants colonized by the endophyte *F. solani* strain K (+F) and/or infested with spider mites (*T. urticae*, +T) compared with the untreated control (–F/–T). Pink indicates upregulation of gene expression levels and a strong up-regulation is indicated in dark red. Light gray indicates downregulation and dark gray indicated strong downregulation of gene expression levels. White indicates the control group. The sample/gene grouping is based on hierarchical clustering (complete linkage algorithm) of the Euclidean sample/gene distances of the differentially expressed genes detected by ANOVA (Tukey’s *post hoc* tests, *P* < 0.05). Values are the mean fold-change compared to relative gene expression in the control plants (–F/–T) of two technical replicates for each of six biological replicates. Expression levels for all target genes were normalized to the geometric mean of ubiquitin and actin expression levels in each sample as a reference. Values and statistically significant differences compared to control samples are presented in Supplementary Table S2. *JIP-21*, *WIPI-II*, *PPO-F/D*, *PI-IIc*, and *LOX-D* are JA-marker genes; *PR-P6* and *PR-1A* are SA-marker genes; *GLU-A/B* and *CHI3/9* are fungal (or herbivore, e.g., whitefly)-marker genes (see relevant references in manuscript).

We found that the expression levels of genes *JIP-21*, *WIPI-II*, *PI-IIc*, and *LOXD*, previously shown to be induced by spider-mites (Kant et al., 2008; Alba et al., 2015; Martel et al., 2015), were also up-regulated in response to spider mite feeding in our study (Figures 4, 5, Supplementary Figure S2 and Supplementary Table S2). Nevertheless, other genes previously reported to be activated or induced by spider mites, such as the *PR-1A*, *PR-P6*, *PPO-D/F*, and *GGPS1* (Kant et al., 2004; Alba et al., 2015) were not consistently altered by the herbivore (Figures 4, 5, Supplementary Figure S2 and Supplementary Table S2).

Tomato colonization of spider mite-infested plants by FsK resulted in a significant further up-regulation in the expression of genes *WIPI-II* and *PPO-D* compared to the un-colonized but spider mite-infested plants (Figures 4, 5 and Supplementary Table S2). No effect was recorded in the expression of the other mite-defense related genes such as *JIP-21*, *PPO-F*, *LOXD*, *PR-1A*, *PR-P6*, and *GGPS1* compared to the respective levels on spider mite-infested plants, except for *PI-IIc* which was shown to be differentially expressed on FsK-colonized plants in response to spider mite feeding depending on the experiment (Figures 4, 5 and Supplementary Table S2). Finally, FsK colonization of spider mite-infested plants significantly affected the expression levels of genes *GLU-A* and *GLU-B* and *CHI9* which were up-regulated on these plants, albeit to similar levels as observed to non-infested plants.

### Tomato Volatiles

**FIGURE 6:**
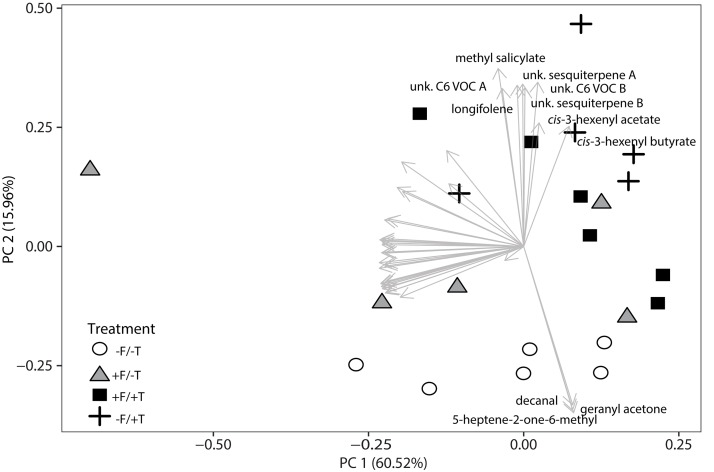
Changes in volatile emissions from tomato plants colonized by the endophyte *F. solani* strain K (+F) and/or infested with spider mites (*T. urticae*, +T). Principal Component Analysis of the autoscaled data of 41 selected volatiles as measured by TD-GC-MS 2 days after spider mite infestation. Analysis was performed on the average peak data. Symbols represent the different biological replicates within each treatment (control plants –F/–T, open circles; FsK*-*colonized plants +F/–T, gray triangles; FsK*-*colonized and spider mite infested plants +F/+T, black squares; and only spider mite infested plants –F/+T, black crosses, 5–6 biological replicates per treatment). Arrows represent the loadings of a single volatile compound. Volatiles that show high loading on PC 2, representing the FsK and spider mite treatment (treatment effect), are labeled by their names.

Having demonstrated significant effects of FsK-colonization of tomato plants on induced direct defense against spider mites, we subsequently investigated whether the endophyte can alter the emission of volatile compounds and thus also contribute to tomato’s indirect defense. We sampled the volatile profiles of FsK-colonized plants with (+F/+T) and without (+F/-T) spider mites. In addition, we sampled volatiles of plants experiencing only herbivory (-F/+T) and plain control plants (-F/-T). This resulted in 41 compounds that were further analyzed. Volatile emissions were different between the treatments when compared with a principal component analysis (PCA) (Figure 6). The first two principal components explained 76.48% combined variance and separated samples between treatments. Control (-F/-T) plants cluster separated from the other treatments and plants experiencing herbivory (-F/+T and +F/+T) grouped together as well. The first principal component can be attributed to between-sample variability, maybe due to sampling effects. The second principal component can be clearly attributed to the effect of the treatments. Compounds with a high loading on the second principal component were highly interesting since they might be the reason for the attraction of the generalist predator *M. pygmaeus* recorded in the olfactometer assays described below.

**FIGURE 7:**
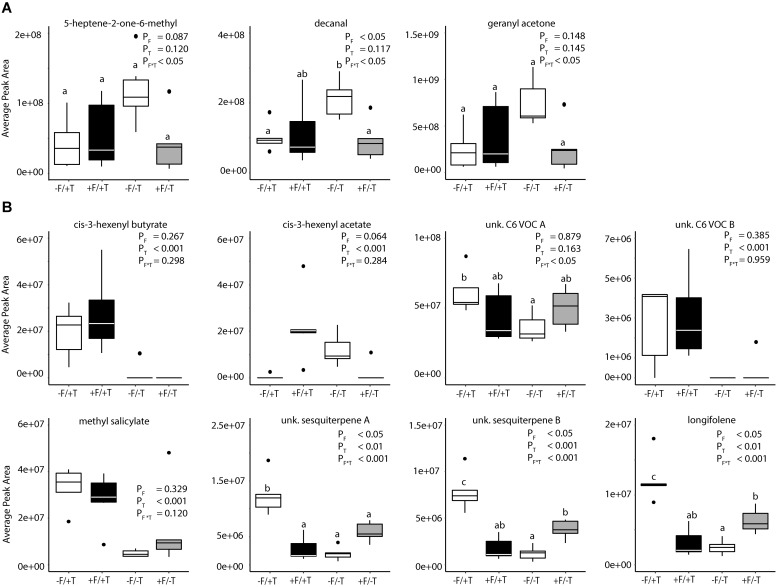
Changes in volatile emissions from tomato plants colonized by the endophyte *F. solani* strain K (+F) and/or infested with spider mites (*T. urticae*, +T). Boxplot of 11 compounds that showed high loading on PC 2. Treatments are: control plants –F/–T; FsK-colonized plants +F/–T; FsK- colonized and spider mite infested plants +F/+T; and only spider mite infested plants –F/+T (5–6 biological replicates per treatment). **(A)** Depicts compounds with negative loadings on PC 2 (treatment effect), while panel **(B)** depicts the compounds with positive loadings. Significant differences between treatments are indicated by different letters by Tukey’s *post hoc* tests after two-way ANOVA: *P* < 0.05.

The analysis of the loadings revealed two distinct groups of compounds. Three (decanal, 5-heptene-2-one-6-methyl and geranyl acetone) had high negative loadings on PC 2 pointing toward the control samples. These compounds (Figure 7A) showed a trend to be suppressed by herbivory or the fungal treatments, even though only significant in the case of decanal (Table 1). Another set of eight compounds (sesquiterpenes, C6 VOCs and MeSA) had a high positive loading on PC 2 pointing toward the herbivory treatment (Figure 7B). Especially, the *cis*-3-hexenyl acetate, C6 VOC B, *cis*-3-hexenyl butyrate and MeSA showed a significant effect of herbivory, whereas the interaction of the endophyte with the herbivore for these compounds was found not significant. On the other hand, a significant interaction effect was recorded for C6 VOC A, the two unknown sesquiterpenes A and B, as well as longifolene (Table 1). The two sesquiterpenes could not be matched to a RI and mass spectra and thus their identity remains elusive. Both sesquiterpenes and longifolene were significantly affected by the presence of the endophyte and the herbivore (Table 1).

**Table 1:** Volatiles emitted by tomato plants according to their calculated Kovats retention index (RI).

		Two-way ANOVA			Tukey HSD *post hoc*					
					
Compound	Calculated RI	Endophyte (F)	Herbivore (T)	Interaction (F^∗^T)	-F/-T			-F/+T		+F/-T
					vs.			vs.		vs.
					-F/+T	+F/-T	+F/+T	+F/-T	+F/+T	+F/+T
5-hepten-2-one-6-methyl^c^	990	0.087	ns	^∗^	0.061	0.060	ns	ns	ns	ns
*cis*-3-hexenyl acetate^b^	1011	0.064	^∗∗∗^	ns						
unk. C6 VOC A	1101	ns	ns	^∗^	0.050	ns	ns	ns	ns	ns
unk. C6 VOC B	1147	ns	^∗∗∗^	ns						
*cis*-3-hexenyl butyrate^b^	1189	ns	^∗∗∗^	ns						
Methyl salicylate^a,b^	1194	ns	^∗∗∗^	ns						
Decanal^b^	1209	^∗^	ns	^∗^	^∗^	^∗^	0.062	ns	ns	ns
unk. sesquiterpene A	1394	^∗^	^∗∗^	^∗∗∗^	^∗∗∗^	0.056	ns	^∗∗^	^∗∗∗^	ns
unk. sesquiterpene B	1407	^∗^	^∗∗∗^	^∗∗∗^	^∗∗∗^	^∗^	ns	^∗∗∗^	^∗∗∗^	0.085
Longifolene^d^	1411	^∗^	^∗∗^	^∗∗∗^	^∗∗∗^	^∗^	ns	^∗∗^	^∗∗∗^	0.099
geranyl acetone^b^	1451	ns	ns	^∗^	0.062	0.077	ns	ns	ns	ns


**FIGURE 8:**
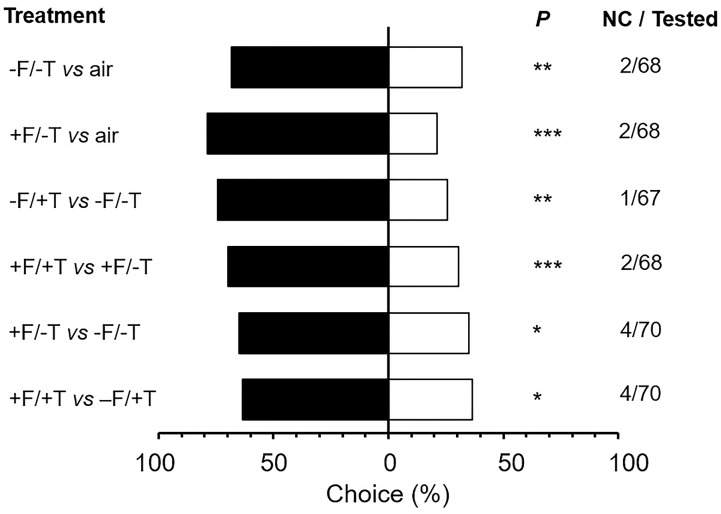
Responses of the generalist predator *Macrolophus pygmaeus* to volatiles emitted by tomato plants colonized by the endophyte *Fusarium solani* strain K (+F) and/or infested with spider mites (*T. urticae*, +T) or clean air (air). One week after seed sowing plants were either inoculated with the endophyte (+F) or not (–F). Subsequently, plants were either infested with 200–300 spider mite females (+T) or not (–T) when 4–5 weeks-old for a period of 2 days. Shown are the percentages of predator females that moved toward the volatile sources in the corresponding choice situations indicated on the left (under ‘Treatment’) in a vertical Y-tube olfactometer. ‘NC/Tested’ indicates the number of individuals that did not make a choice over the total number of tested individuals (67–70 individuals depending on the treatment combination). Significant differences are indicated by asterisks after a χ^2^ test: ^∗^*P* < 0.05, ^∗∗^*P* < 0.01, ^∗∗∗^*P* < 0.001.

*^b^Adams (1995); ^c^Lucero et al. (2003); ^d^Kant et al. (2004). Compounds presented are from the PCA in Figure 7 and the same as shown in Figure 8 and were identified by comparison to an authentic standard^a^ or tentatively identified by comparison to RI values in the literature^b,c,d^ when possible. Two-way ANOVA (df = 1) with Endophyte (F) and Herbivore (T) as factors was performed on the average peak area. Tukey HSD was carried out as *post hoc* analysis for compounds showing a significant interaction (F^∗^T) effect. P-values marked with an asterisk represent ^∗^P < 0.05, ^∗∗^P < 0.01, ^∗∗∗^P < 0.001, ns represent values P > 0.1. For P-values 0.1 > P > 0.05 the values are shown.*

Besides the 11 compounds depicted in the PCA, another 30 compounds were also identified for which two-way ANOVA revealed no significant interaction effect (Supplementary Table S3). Of these 30 compounds, 13 were found to be significantly affected by the presence of spider mites only, and none of the endophyte suggesting that, in total, 17 of the sampled volatiles were identified to be herbivore-specific (Table 1 and Supplementary Table S3).

### Predator Choice

To test whether the differences in volatile emissions caused by FsK colonization could be functionally significant in indirect defense, we used a Y-tube olfactometer choice test with the generalist predator *M. pygmaeus*. Only a few predators (1.5–5.7% depending on the treatment, χ^2^ = 3.84; df = 5; *P* = 0.573) used in the olfactometer assays did not make a choice within the time frame of 12 min and, thus, were excluded from data analysis (Figure 8). *M. pygmaeus* females preferred volatiles from plants (either FsK-colonized or not) over clean air (Figure 8; -F/-T vs. air: χ^2^ = 8.73; *P* = 0.003; +F/-T vs. air: χ^2^ = 21.88; *P* < 0.001). In addition, females preferred volatiles from spider mite-infested plants that were either FsK-colonized or not (Figure 8; -F/+T vs. -F/-T: χ^2^ = 10.24; *P* = 0.001; +F/+T vs. +F/-T: χ^2^ = 15.51; *P* < 0.001). Volatiles from FsK-colonized plants were more attractive than those from un-colonized plants, either when these were spider mite-infested or not (Figure 8; +F/-T vs. -F/-T: χ^2^ = 6.06; *P* = 0.014; +F/+T vs. -F/+T: χ^2^ = 4.90; *P* = 0.027).

## Discussion

In the present study, we tested to which extent the colonization of plants by the endophytic fungus *F. solani* strain K affects tomato responses against spider mites. We found that defense-related genes were differentially expressed on colonized plants. In accordance, spider mites laid a lower number of eggs on FsK-colonized plants. We also observed that feeding damage inflicted by spider mites was lower on FsK-colonized plants compared to control plants. However, plant biomass was increased in the presence of the endophyte and the spider mites. Therefore, we argue that there are indications for the existence of plant growth promotion capabilities in the endophyte but also for the expression of herbivore-induced compensatory growth in tomato. Finally, we also recorded substantial differences in the volatiles emitted by endophyte-colonized plants that were either spider mite-infested or not. As a result, tomato indirect defense was enhanced and the zoophytophagous predator *M. pygmaeus*, a natural enemy of spider mites, was able to identify spider mite-infested but also FsK-colonized plants in all colonization/infestation-type combinations.

### Tomato Colonization by the Endophyte Leads to Reduced Spider Mite Performance and Increased Plant Biomass

Plant-mediated effects of beneficial soil microbes on spider mites have been scarcely addressed so far. To the best of our knowledge, AMF are the most, if not the main, group of soil microbes that have been studied in this regard. In contrast to what is generally known about the negative effects of several AMF on generalists and mesophyll feeders (Hartley and Gange, 2009; Pineda et al., 2010; Shikano et al., 2017), the two-spotted spider mite is a generalist parenchyma cell-content feeder but has been previously shown to be positively affected by AMF in symbiosis with different plant species. For example, *T. urticae* performance was shown to be enhanced on common bean plants *Phaseolus vulgaris* by the AMF *Glomus* *mosseae* and/or the nitrogen-fixing bacteria *Azotobacter* *chroococcum* (Hoffmann et al., 2009; Hoffmann et al., 2011; Khaitov et al., 2015). In these cases, the benefit for the herbivore was attributed to the improved nutritional value of the plant tissue which correlated to enhanced uptake of P and K by AMF and improved N uptake by *A. chroococcum*. Another study, however, that explored the effects of four different AMF species belonging to different genera, found that spider mite (*T. urticae*) performance in *Lotus japonicus* was differentially impacted depending on the AMF species. In the same study, defense-related compounds were also differentially altered by the various AMF species, suggesting that AMF effects on aboveground herbivorous mites and plant responses are species-specific and variable (Nishida et al., 2010). Nevertheless, symbioses of soil microbes with other plants such as tomato and their interactions with aboveground mites remain unexplored.

Our study is a first report of a beneficial soil endophytic fungus that negatively affects spider mite performance in tomato. *Fusarium solani* strain K has been previously shown to mediate tomato systemic resistance against other foliar organisms, such as the pathogen *Septoria lycopersici* (Kavroulakis et al., 2007) as well as against the feeding damage inflicted by the zoophytophagous predator *N. tenuis* (Garantonakis et al., 2018). Similarly, a pathogenic *Fusarium* species in tomato (*F.* *oxysporum* f. sp. *lycopersici* race 1) was shown to induce plant resistance against spider mites that led to reduced oviposition on a *Fusarium*-susceptible tomato line, however, when plants were also water-stressed (Jongebloed et al., 1992). The exact mechanism(s) involved in FsK-mediated resistance to foliar organisms is not known. Yet, there is evidence toward the involvement of the ethylene and JA signaling pathways in FsK-mediated resistance to *N. tenuis* feeding (Garantonakis et al., 2018). Furthermore, an earlier study has shown that tomato resistance to a root pathogen by FsK was only expressed in the presence of intact ethylene signaling pathway, independently of JA (Kavroulakis et al., 2007). In the latter study, root colonization by FsK did not induce elevated ethylene production in the root and aerial parts whereas SA-mediated responses in roots (but not in leaves) were suppressed. This data, although limited, suggest the involvement of all three important signaling pathways, which also mediate plant responses to herbivory, in the FsK-resistance cases reported so far. Nevertheless, a thorough study of the phytohormone accumulation in the aerial parts of FsK-colonized plants is required to cast light on the mechanisms involved in FsK-mediated tomato resistance against spider mites.

When it comes to herbivory the net benefit of a mutualistic relationship among soil microbes and plants depends on the trade-off between microbe-induced plant defenses versus plant nutritional quality or quantity alteration (Pozo and Azcón-Aguilar, 2007; Gehring and Bennett, 2009; Kempel et al., 2010; Pineda et al., 2010; Shikano et al., 2017). In the present study, however, putative mechanisms involved in FsK-mediated resistance against spider mites were shown to be related to both defense elicitation and plant growth promotion by FsK. Endophytes in general have been shown to promote plant growth and to affect resource allocation in ways that impact the host’s ability to compensate for herbivory (Vessey, 2003; Hartley and Gange, 2009; Rodriguez et al., 2009; Bever et al., 2013; Dupont et al., 2015). We herein recorded shoot but not root growth promotion by FsK in 5–6 weeks-old tomato plants in the absence of spider mites (Figure 3). This effect was not evident for plants that were 10 days younger (data not shown). On the other hand, compensatory plant growth in response to herbivory was also recorded for the endophyte or the spider mites alone whereas root weight was further stimulated in response to their combined effect (Figure 3). Herbivore-induced plant growth may be promoted in the plant’s attempt to compensate for herbivory and depends on herbivore characteristics and herbivore density (Järemo and Palmqvist, 2001). For example, photosynthetic activity was shown to be stimulated in cucumber and chrysanthemum at low populations of spider mites (Tomczyk et al., 1991). Although additional plant growth and developmental factors would be needed to evaluate the biological significance of this observation, it is clear that FsK further stimulates plant growth, acting complementary to plant responses to spider mites alone. Spider mites were adversely impacted on FsK-colonized plants (Figures 1, 2), suggesting the absence of nutritional benefits and/or that defense induction outcompetes the putative benefits of improved nutrition to the herbivore. It should be noted, however, that leaflet area was found not to be different among FsK-colonized and un-colonized plants suggesting that spider mites had no access to additional food supply on either plant.

The number of live spider mites on plants was not different among FsK-colonized and control plants (Figure 1), suggesting that recorded differences can be attributed to plant responses affecting spider mite reproduction. Spider mites are known to be sensitive mainly to JA- and to a lesser extent to SA-mediated defenses (Li et al., 2002; Kant et al., 2008; Alba et al., 2015; Ataide et al., 2016; Villarroel et al., 2016). In accordance, both JA and SA defense marker genes were shown to be up-regulated in the presence of FsK in spider mite-infested plants in the present study (Figures 4, 5 and Supplementary Table S2). Hence, we may assume that JA and SA effectual defenses are induced in FsK-colonized plants against spider mites. JA-activation is common in other mutualistic symbioses such as with PGPR and AMF. In these cases, symbiotic plants show a JA-related ‘primed’ state of defense that results in plant resistance to chewing herbivores and necrotrophic pathogens (Pineda et al., 2010; Zamioudis and Pieterse, 2012; Pieterse et al., 2014; Shikano et al., 2017). Hence, in addition to the clarification of JA and SA involvement, whether priming is also involved in FsK-mediated resistance to spider mites is yet to be determined.

### Tomato Defense-Gene Expression Is Altered by the Endophyte

In tomato, JA signaling is the most important regulator of spider-mite induced defenses (Kant et al., 2015; Martel et al., 2015). Similarly to previous reports, we also recorded the up-regulated expression of genes *JIP-21*, *WIPI-II* [or *PI-IIf*, see Alba et al. (2015)], *LOXD* and *PI-IIc* in response to spider mite feeding (Figures 4, 5, Supplementary Figure S2 and Supplementary Table S2) that are known to mark JA defenses in tomato (Kant et al., 2004; Alba et al., 2015; Martel et al., 2015). It should be noted, however, that the KOP spider mite strain used in the present study is a tomato-adapted line previously shown to resist JA defenses in tomato (Ament et al., 2004; Kant et al., 2008). Importantly, KOP mites have been shown to perform equally well on *def-1* and *PS* cultivars, a JA biosynthesis mutant and a transgenic tomato plant *35S::prosystemin*, respectively, and on WT plants (Kant et al., 2008). Hence, despite the ability of KOP spider mites to induce JA-dependent responses, as also verified in our study, we expected these mites to display a resistant phenotype on our tomato plants. Indeed, our results show that KOP mites produce a similar or slightly lower number of eggs compared to previous reports (Ament et al., 2004; Kant et al., 2008) that decreased to approx. 3.5 eggs/female/day on FsK-colonized plants in our study (Figure 1). On the other hand, other genes such as *PR-1A*, *PR-P6*, *PPO-D/F*, and *GGPS1* that have been previously shown to be up-regulated by other spider-mite strains [KMB or *T. urticae* Santpoort-2 in Kant et al. (2008) and Alba et al. (2015), respectively] were not shown to be consistently affected in our study (see for example, *PR-1A* expression in Figures 4, 5, Supplementary Figure S2 and Supplementary Table S2). This could be attributed to differences in our experimental set-up with previous studies (e.g., different tomato cultivar and spider mite line, soil-less experimental plants in our study). It also indicates the limitations of marker genes to deduce conclusions as regards to the molecular mechanisms that govern multi-partite interactions, such the ones studied here. Hence, the dynamics that result in the reduced performance of spider mites we were able to show in independent experimental set-ups require a more detailed analysis. On the other hand, *PPO* genes encode proteins that normally reduce amino acid availability from ingested plant tissue in the gut of herbivores. As such, these compounds are believed to have little or no effect on herbivores with acid guts such as spider mites (Erban and Hubert, 2010; Carrillo et al., 2011; Martel et al., 2015). This may explain why spider-mite infestation may not induce a consistent induction of these genes.

Tomato colonization by the endophyte resulted in differential expression of defense-related genes (Figures 4, 5, Supplementary Figure S2 and Supplementary Table S2). As expected, genes *GLU-A* and *CHI9* that are typically induced against beneficial and pathogenic fungi or other herbivores [e.g., whiteflies, Puthoff et al. (2010)] were shown to be up-regulated in FsK-colonized plants. Up-regulation was also recorded for *PI-IIc* and *PR-1A* whereas *PPO-F* was down-regulated on FsK-colonized compared to un-colonized plants. In the presence of the herbivore, FsK retains the capability to induce these genes as compared to uncolonized plants. In addition, FsK colonization led to a significant further increase of the transcript levels of *WIPI-II*. These results correlate well with the significant decrease in spider mite oviposition recorded on these plants in our study (Figure 1) and to the rather conserved mechanism reported for microbe-induced JA-mediated defenses in response to herbivory (Pineda et al., 2010; Shikano et al., 2017). KOP is a tomato-resistant spider mite line that performs equally well on JA-defended and WT plants (Kant et al., 2008) whereas spider mites (*T. urticae*) have been shown to be negatively impacted by JA defenses up to the endogenous JA-IIe levels of around 10 ng.gFW^-1^ (Ataide et al., 2016). Most possibly this plateau was not reached on spider mite-infested plants thus enabling FsK to prime or, further induce, effectual JA defenses such as *WIPI-II* expression against KOP mites. This effect was also consistently observed in *WIPI-II* expression levels in our second independent experimental set-up (Supplementary Figure S2).

In tomato, the increasing emission of certain volatiles such as the homoterpene (E,E)-4,8,12-trimethyltridecane-1,3,7,11-tetraene (TMTT) and methyl salicylate (MeSA) in response to spider mite feeding is well-documented (Ament et al., 2004, 2006; Kant et al., 2004; Kant et al., 2008; Nagaraju et al., 2012). TMTT has been shown to play a prominent role in indirect defense since it is attractive to predators of spider mites (Dicke et al., 1990, 1998, 1999; Ament et al., 2004; Sarmento et al., 2011) but we were unable to detect it in our analysis. This may be attributed to the sampling time (volatiles were collected 2 days after spider mite feeding in our study), whereas Kant et al. (2004) reported that a 3-day delay is required for indirect defense mounting. This compound is synthesized by the precursor molecule geranylgeranyl diphosphate by the activity of the enzyme geranylgeranyl diphosphate synthase. Gene *GGPS1* encoding this biosynthetic enzyme is known to be induced by *T. urticae* (Kant et al., 2004; Ament et al., 2006). In the present study, we could not detect consistently this up-regulation of *GGPS1* expression levels at 4 days after spider mite feeding (Figures 4, 5, Supplementary Figure S2 and Supplementary Table S2). The same was reported by Martel et al. (2015) for plants infested with spider mites for only 1 day. It is plausible that a time-delay for mounting an indirect defense in response to spider mite feeding in tomato is required, and may explain the inconsistencies both in the gene expression levels and the lack of TMTT emission in our study (Figures 4, 5, Table 1 and Supplementary Table S3) Nevertheless, other volatiles were found to be emitted by FsK-colonized plants corroborating the hypothesis that FsK may also mediate indirect tomato defense to attract natural enemies.

### Indirect Tomato Defense Against Spider Mites Is Enhanced by the Endophyte

Beneficial soil microbes are known to be capable of affecting indirect plant defense by mediating the expression of plant traits that impact natural enemies (Rasmann et al., 2017). Volatile emission is one such trait that can be directly impacted by soil microbes through their impact on plant metabolites. On the other hand, increased volatile emission and eventually indirect defense, could result from microbe-induced promotion of growth rate, plant vigor or size when linked to increased prey density and volatile emission. In contrast to microbe-mediated effects to plant morphology, the effects of beneficial soil microbes on plant volatile production have been better documented so far (Rasmann et al., 2017). As with direct defenses, however, most of our current knowledge derives from studies on AMF-activated effects on volatiles (e.g., Guerrieri et al., 2004; Fontana et al., 2009; Babikova et al., 2014) and the impact of PGPR, rhizobia and endophytic fungi have been scarcely addressed so far. Beneficial soil microbes are generally assumed to affect constitutive and inducible volatile production in response to herbivory but the outcome of these tri-trophic interactions seems to be vastly species-specific and context dependent. Considering spider mites, only one study reporting the enhanced production of the sesquiterpenes β-omicene and β-caryophyllene induced by *T. urticae* on AMF-colonized bean plants that resulted in an enhanced attraction of the predatory mite *P. persimilis* toward these plants has been reported so far (Schausberger et al., 2012). Hence, our knowledge on microbe-mediated effects on indirect plant defense to spider mites is limited, even more on such effects of endophytic fungi on herbivores in general and, spider mites in particular.

Very little is known about the effects of endophytic fungi on plant volatile production [mainly focused on *Trichoderma* species, e.g., Battaglia et al. (2013), Coppola et al. (2017), Contreras-Cornejo et al. (2018)] and to the best of our knowledge, no previous study has ever explored how plant colonization by an endophytic fungus mediates the volatile-regulated attraction of insect predators to spider mite-infested plants. We herein show that the generalist predator *M. pygmaeus* was attracted to FsK-colonized plants irrespectively of the presence of spider mites (Figure 8). This predator is known to be attracted by volatiles emitted by tomato plants in response to herbivory (e.g., by *Tuta* *absoluta* or *Bemisia* *tabaci*) (Lins et al., 2014; De Backer et al., 2015; De Backer et al., 2017). From the volatile blend emitted by *T. absoluta*-infested tomato plants, (*E*)hex-2-enal, 2-carene, α-pinene, β-phellandrene, hexanal, and linalool were found to evoke positive attraction in *M. pygmaeus* (De Backer et al., 2017). Our volatile analyses clearly show that the volatile blends of tomato plants are affected by the presence of the endophyte and the same holds for the volatile blends emitted by spider mite-infested plants compared to control plants (Figure 6, Table 1 and Supplementary Table S3).

The TMTT and MeSA constitute the most abundant volatiles emitted by tomato in response to spider mite feeding (Ament et al., 2004; Ament et al., 2006). Nevertheless, as noted earlier, TMTT was not detected in our volatile sampling 2 days after spider mite infestation, which is surprising since *GGPS1* was also not expressed after another 4 days. On the other hand, JA-dependent MeSA emission by tomato plants was significantly increased in response to spider mite feeding in our study (Figure 7 and Table 1) in accordance with a previous report showing a two-fold increase in MeSA emission in response to KOP mites feeding (Kant et al., 2008). In addition, another 16 volatiles were significantly affected by the presence of spider mites alone in our study (Table 1 and Supplementary Table S3) suggesting that these compounds are spider mite-specific. Of these volatiles, β-myrcene is known to increase upon spider mite infestation in tomato, although not always significantly (Kant et al., 2004). In addition, β-caryophyllene and β-phellandrene were found to be significantly affected by spider mite infestation in our study although previously reported to be produced constitutively by tomato plants, and not induced by spider mites (Kant et al., 2004; Sarmento et al., 2011). In contrast, the induction of *trans*-nerolidol and *trans*-β-ocimene by spider mites, shown to be JA-dependent by Ament et al. (2004), was not significantly affected by spider mites in our study. Finally, α-terpinene, recently suggested for its putative role in the attraction of *M. pygmaeus* to tomato (Cortés et al., 2016) was also found herein to be significantly affected by spider mites.

The fact that the predators were able to identify FsK-colonized from un-colonized plants even when these were infested with spider mites (Figure 8) suggests that endophyte effects on volatile blend alteration are functionally important for tritrophic interactions. Identifying the specific volatiles mediating the predator attraction to FsK-colonized plants might be difficult since arthropods are known to respond to volatile blends, rather than specific chemicals (Gols et al., 2011; van Wijk et al., 2011). Nevertheless, we should note that endophyte colonization of tomato plants led to increased emissions of one sesquiterpene (unknown sesquiterpene B, calculated RI: 1407, Figure 7 and Table 1) and longifolene, whereas the emission of decanal was significantly suppressed (Figure 7 and Table 1). On the other hand, spider mite infestation of FsK-colonized plants led to a decreased emission of unknown sesquiterpenes A and B, as well as longifolene (Figure 7 and Table 1). Notably, these plants displayed a stronger attraction of the predators over uninfested, FsK-colonized plants (Figure 8). Taken together, it would be interesting to further assess the role of the two sesquiterpenes as well as decanal and longifolene in shaping tomato indirect defense.

Collectively, our data support the hypothesis that the endophytic fungus *F. solani* strain K alters tomato responses to spider mites to the benefit of the plant. Putative mechanisms involved are shown herein to vary between defense induction and/or priming to plant growth promotion and tolerance. In the present study, we observed these mechanisms to be separately displayed. Hence, the temporal dynamics of FsK-related resistance/tolerance mechanisms in tomato should be further explored, following non-targeted transcriptomics approaches as well. Moreover, a detailed study is required to cast light on the dynamics and interactions of phytohormones underlying the endophyte’s role in shaping tomato-spider mite interactions. Besides direct defense activation, FsK was also shown to enhance indirect tomato defense. The putative adaptive value of predator attraction to FsK-colonized plants lies in the fact that plants might have to invest less in direct defense activation, provided that volatile production does not entail major energetic costs. On the other hand, the attraction of mirids to the well-defended FsK-colonized plants might impose no harm to the predators when, similarly to AMF-colonized plants (Pineda et al., 2010; Shikano et al., 2017), FsK-colonized plants also display susceptibility to sucking insects (e.g., aphids and whiteflies). Hence, to draw safe conclusions about the protective role of FsK in tomato, it is imperative to assess its effects against other herbivores as well, also in relation to its impact on root-feeding organisms (e.g., arthropods or nematodes). Ultimately, the net benefit of FsK-colonization for the plant and its potential as a novel tool in spider mite control should be confirmed by studying the effects of FsK-mediated resistance on plant fitness and reproductive output. Finally, it must be noted that our experiments were performed with plants grown on a sand mixture with vermiculite. Hence, some of the differences observed between the present study and previous works may be related to the absence of other soil microbes in our study that in different settings (i.e., when plants are grown in soil) could interact with the endophyte and/or impact gene expression and volatile emission in aboveground plant parts (Benítez et al., 2017).

## Author Contributions

MP, GB, and KP conceived and designed the experiments. ML, MP, DP, and MA performed the experiments. GB, MP, KP, NK, and AW analyzed the data. MP wrote the manuscript with input from KP, GB, and AW. All authors read, edited, and approved the final manuscript.

## Conflict of Interest Statement

*Fusarium* *solani* FsK is patented (20070100563/1006119, issued by the Industrial Property 319 Organization to NK and KP). The remaining authors declare that the research was conducted in the absence of any commercial or financial relationships that could be construed as a potential conflict of interest.
