# Supplementary material for: The Beneficial Endophytic Fungus Fusarium solani Strain K Alters Tomato Responses Against Spider Mites to the Benefit of the Plant
Source: Front Plant Sci. 2018 Nov 6;9:1603. doi: 10.3389/fpls.2018.01603 (PMC6232530; doi:10.3389/fpls.2018.01603)
Supplement: Supplementary file 1 [file Data_Sheet_1.pdf]

Supplementary Material

The beneficial endophytic fungus *Fusarium solani* strain K alters tomato responses against spider mites to the benefit of the plant

Maria L. Pappas*, Maria Liapoura, Dimitra Papantoniou, Marianna Avramidou, Nektarios Kavroulakis, Alexander Weinhold, George D. Broufas, Kalliope K. Papadopoulou

*Correspondence: Maria L. Pappas mpappa@agro.duth.gr

Supplementary Figures

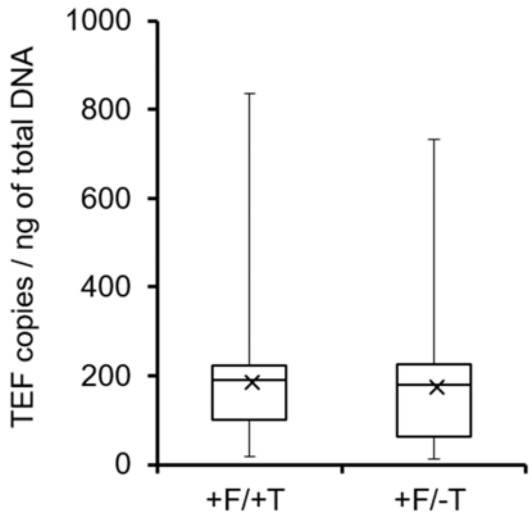

Figure S1. Effect of spider mite infestation of tomato plants on fungal (FsK) colonization within root tissues. Quantification of fungal colonization within root tissues by qPCR using primers specific for *Fusarium Tef-1a* gene (primary axis). Values are normalized to ng of total DNA isolated. Data are means of two technical replicates for each of seven biological replicates. Each box plot thick horizontal line shows the median, 'x' the sample mean, upper and lower box boundaries the quartiles, and whiskers the extreme values within a category. Roots were harvested from plants used for the gene expression analysis. No significant differences in FsK colonization were recorded among plants infested with spider mites (+F/+T) or not (+F/-T) after Student's *t*-test.

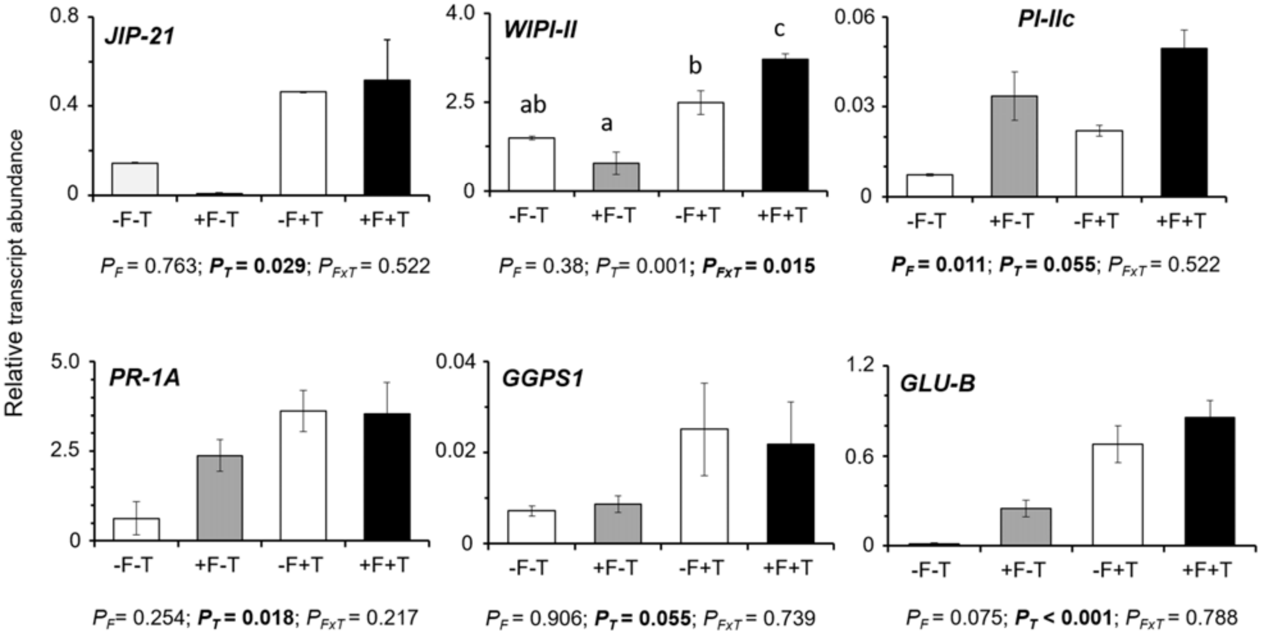

Figure S2. Effects on the transcript levels of defense marker genes in tomato plants colonized by the endophyte *Fusarium solani* strain K (+F) and/or infested with spider mites (*Tetranychus urticae*, +T) compared with the untreated control (-F/-T). Values are the average ± SE of two technical replicates for each of three biological replicates of a second, independent experiment to the one presented in Fig. 4 and Table 1. Expression levels for all target genes were normalized to the geometric mean of ubiquitin and actin expression levels in each sample as a reference. Two-way ANOVA (α=0.05) *P*-values are shown for each gene under the graph. Significant *P*-values (< 0.05) are indicated in bold: P_F, probability value for the endophyte (FsK) effect; P_T, probability value for the herbivore (T) effect; P_{F*T}, probability value for FsK x T interaction. In the case of significant interaction ($P_{F*T} < 0.05$) significant differences between treatments are indicated by different letters by Tukey's post-hoc tests after two-way ANOVA: $P < 0.05$. *JIP-21*, *WIPI-II*, and *PI-IIc* are JA-marker genes; *PR-1A* is a SA-marker gene; *GLU-B* is a fungal (or herbivore e.g. whitefly)-marker gene and *GGPS1* has been reported as responsive to spider mite infestation in tomato (see relevant references in manuscript).

Supplementary Tables

Table S1. Primers used for gene expression analysis by qPCR

Target gene	GenBank (GB) accession	Target Gene	Primer sequence 5' to 3'
JIP-21	AJ295638.1	JIP-21_F	ACTCGTCCTGTGCTTTGTCC
Jasmonate-inducible protein 21		JIP-21_R	CCCAAGAGGATTTTCGTTGA
WIPI-II	AY129402.1	PI-IIf_F	GACAAGGTACTAGTAATCAATTATCC
Wound-induced Proteinase Inhibitor IIf		PI-IIf_R	GGGCATATCCCGAACCCAAGA
PI-IIc	X94946.1	PI-IIc_F	CAGGATGTACGACGTGTTGC
Proteinase Inhibitor IIc		PI-IIc_R	GAGTTTGCAACCCTCTCCTG
PPO-F	AK247126.1	PPO-F_F	CGGAGTTTGCAGGGAGTTATAC
Polyphenol-oxidase-F		PPO-F_R	TTGATCTCCACACTTTCAATGG
PPO-D	Z12836.1	PPO-D_F	GCCCAATGGAGCCATATC
Polyphenol-oxidase-D		PPO-D_R	ACATTCGATCCACATTGCTG
LOXD		LOXD-F	F: 5'-CCTGAAATCTATGGCCCTCA-3'
Lipoxygenase D		LOXD-R	R: 5'-ATGGGCTTAAGTGTGCCAAC-3'
PR-1A	AJ011520	PR1a_F	F: 5'-TCTTGTGAGGCCCAAAATTC-3'
Acidic pathogenesis-related protein 1a		PR1a_R	R: 5'-ATAGTCTGGCCTCTCGGACA-3'
PR-P6	Y08804.1	PR-P6_F	GTACTGCATCTTCTTGTTTCCA
Pathogenesis related protein P6		PR-P6_R	TAGATAAGTGCTTGATGTGCC
GGPS1	NM_001247158.1	GGPS-I_F	GGCAGATTGTGGACTTGGCGA
Geranylgeranyl pyrophosphate synthase 1		GGPS-I_R	CTCATTCGCTCCACATCAACC
GLU-A	M80604	GLUA-F	F: 5'-GGTCTCAACCGCGACATATT-3'
Acidic β-1,3-glucanase		GLUA-R	R: 5'-CACAAGGGCATCGAAAAGAT-3'
GLU-B	M80608	GLUB-F	F: 5'-TCTTGCCCCATTTCAAGTTC-3'
Basic β-1,3-glucanase		GLUB-R	R: 5'-TGCACGTGTATCCCTCAAAA-3'

CHI3	Z15141	CHI3-F	F: 5’-TGCAGGAACATTCACTGGAG-3’
Acidic chitinase		CHI3-R	R: 5’-TAACGTTGTGGCATGATGGT-3’
CHI9	Z15140	CHI9-F	F: 5’-GAAATTGCTGCTTTCCTTGC-3’
Basic chitinase		CHI9-R	R: 5’-CTCCAATGGCTCTTCCACAT-3’
UBQ	BT012724.1	LeUbl-F	AAGATGGAAGGACTCTGG
Ubiquitin		LeUbl-R	TCACAACACATCACAAGGTC
Tef-1a		Tef1a_F	CCCCTCCAGGATGTCTACAA
Nectria haematococca *translation elongation factor 1a*		Tef1a_R	GGAAGACCCTCAGTGAGCTG

Table S2: Fold-change of tomato defense-gene expression mean values (± SE) in plants colonized with FsK (+F) and/or infested with spider mites *(Tetranychus urticae,* +T*)* compared to the untreated control (-F/-T). Two-way ANOVA *P*-values lower than 0.05 are shown in bold and indicate significant effects. P_F, probability value for the endophyte (FsK) effect; P_T, probability value for herbivore (T) effect; P_{F*T}, probability value for FsK x T interaction. In the case of significant interaction (P_{F*T} < 0.05) significant differences between treatments are indicated by different letters by Tukey's post-hoc tests after two-way ANOVA: $P < 0.05$.

Gene	Treatment				*P*-values
	A<br>**-F/-T**	B<br>**+F/-T**	C<br>**-F/+T**	D<br>**+F/+T**	
JIP-21	1<br>±0.46	0.24<br>±0.12	9.26<br>±4.31	10.64<br>±2.92	P_F = 0.907; $\mathbf{P_T}$ **= 0.001**;<br>P_{FxT} = 0.686
WIPI-II	1<br>±0.37a	0.71<br>±0.28a	3.68<br>±0.68b	15.03<br>±4.38c	P_F = 0.022; P_T = 0.001;<br>$\mathbf{P_{FxT}}$ **= 0.016**
PI-IIc	1<br>±0.29a	2.85<br>±0.68a	5.07<br>±0.80b	2.01<br>±0.46a	P_F = 0.288; P_T = 0.009;<br>$\mathbf{P_{FxT}}$ **< 0.001**
PPO-F	1<br>±0.22a	0.28<br>±0.06b	0.16<br>±0.06b	0.51<br>±0.08b	P_F = 0.154; P_T = 0.020;<br>$\mathbf{P_{FxT}}$ **< 0.001**
PPO-D	1<br>±0.24ab	0.78<br>±0.21b	0.47<br>±0.08b	1.3<br>±0.24a	P_F = 0.206; P_T = 0.794;<br>$\mathbf{P_{FxT}}$ **= 0.042**
LOXD	1<br>±0.13	0.69<br>±0.15	1.38<br>±0.13	1.32<br>±0.12	P_F = 0.161; $\mathbf{P_T}$ **= 0.001**;<br>P_{FxT} = 0.347
PR-1A	1<br>±0.03a	2.1<br>±0.10b	0.65<br>±0.01a	1.54<br>±0.02a	P_F =0.043; P_T = 0.047;<br>$\mathbf{P_{FxT}}$**=0.027**
PR-P6	1<br>±0.52	2.98<br>±1.69	3.68<br>±1.07	1.54<br>±0.43	P_F = 0.966; P_T = 0.626;<br>P_{FxT} = 0.067
GGPS1	1<br>±0.31	2.58<br>±0.53	1.38<br>±0.21	2.56<br>±0.47	P_F = 0.054; P_T = 0.853;<br>P_{FxT} = 0.811
GLU-A	1<br>±0.07	1.98<br>±0.08	0.74<br>±0.08	1.96<br>±0.03	$\mathbf{P_F}$ **= 0.041**; P_T = 0.478;<br>P_{FxT} = 0.740
GLU-B	1<br>±0.06	2.79<br>±0.18	0.19<br>±0.01	1.59<br>±0.05	$\mathbf{P_F}$ **= 0.026**; P_T = 0.146;<br>P_{FxT} = 0.769
CHI3	1<br>±0.52	2.70<br>±1.06	1.44<br>±0.25	1.6<br>±0.32	P_F = 0.152; P_T = 0.603;<br>P_{FxT} = 0.235
CHI9	1<br>±0.43	2<br>±0.69	0.28<br>±0.07	1.24<br>±0.35	$\mathbf{P_F}$ **= 0.039**; P_T = 0.110;<br>P_{FxT} = 0.957

Table S3. Volatiles emitted by the tomato plants, in addition to the volatiles presented in Table 1, according to their measured retention time and calculated Kovats retention index (RI). Compounds were identified by comparison to an authentic standard[a] or tentatively identified by comparison to RI values in the literature[b.c.d.e] when possible. The *P*-values have been calculated with two-way ANOVA (df = 1) with *Endophyte* (F) and *Herbivore* (T) and their interaction as factors. *P*- values marked with an asterisk represent $P < 0.05$ (*).

		Two-way ANOVA		
**Compound**	**Calculated RI**	**Endophyte (F)**	**Herbivore (T)**	**Interaction (F*T)**
hexanal [b]	803	ns	ns	ns
cis-3-hexenol[a.b]	867	0.086	ns	ns
α-thujene[b]	928	ns	ns	ns
α-pinene[a.b]	935	ns	ns	ns
unk. monoterpene A	952	ns	ns	ns
unk. monoterpene B	975	ns	0.071	ns
β-pinene[b]	979	ns	ns	ns
β-myrcene[b]	992	ns	*	ns
carene isomer[d]	999	ns	0.053	ns
carene isomer[d]	1004	ns	*	ns
α-phellandrene[b]	1006	ns	*	ns
unk. monoterpene C	1010	ns	ns	ns
α-terpinene[b]	1019	ns	*	ns
unk. monoterpene D	1025	ns	*	ns
β-phellandrene[b]	1035	ns	*	ns
trans-*β*-ocimene[b]	1053	ns	0.066	ns
γ-terpinene[b]	1062	ns	*	ns
α-terpinolene[b]	1087	ns	*	ns
unk. monoterpene E	1103	ns	ns	ns
DMNT[d]	1117	ns	*	ns
trans-2-caren-4-ol[e]	1179	ns	ns	ns
unk. sesquiterpene C	1334	ns	0.055	ns
δ-elemene[d]	1338	ns	0.054	ns
β-elemene[d]	1390	ns	*	ns
β-caryophyllene[a.d]	1421	ns	*	ns
β-farnesene[b]	1444	ns	0.057	ns
α-caryophyllene[b]	1458	ns	*	ns
γ-muurolene[b]	1483	ns	*	ns
cis-nerolidol[d]	1530	ns	0.055	ns
trans-nerolidol[d]	1562	ns	0.081	ns

[b]Adams (1995); [c]Lucero et al. (2003); [d]Kant et al. (2004); [e]Yousefzadi et al. (2011)

References

Adams, R.P. (1995). *Identification of Essential Oil Components by Gas Chromatography/Mass Spectroscopy*.

Kant, M.R., Ament, K., Sabelis, M.W., Haring, M.A., and Schuurink, R.C. (2004). Differential timing of spider mite-induced direct and indirect defenses in tomato plants. *Plant Physiology* 135(1)**,** 483-495. doi: 10.1104/pp.103.038315.

Lucero, M.E., Estell, R.E., and Fredrickson, E.L. (2003). The essential oil composition of *Psorothamnus scoparius* (A. Gray) Rydb. *Journal of Essential Oil Research* 15(2)**,** 108-111. doi: 10.1080/10412905.2003.9712083.

Yousefzadi, M., Heidari, M., Akbarpour, M., Mirjalili, M.H., Zeinali, A., and Parsa, M. (2011). In vitro cytotoxic activity of the essential oil of *Dorema ammoniacum* D. *Don. Middle East J Sci Res* 7(4)**,** 511-514.
